# Budgerigars adopt robust, but idiosyncratic flight paths

**DOI:** 10.1038/s41598-020-59013-3

**Published:** 2020-02-13

**Authors:** Debajyoti Karmaker, Julia Groening, Michael Wilson, Ingo Schiffner, Mandyam V. Srinivasan

**Affiliations:** 1 Queensland Brain Institute, The University of Queensland, St Lucia, QLD Australia; 2 School of Natural Sciences, Bangor University, Gwynedd, Wales UK; 3 School of Information Technology and Electrical Engineering, The University of Queensland, St Lucia, QLD Australia

**Keywords:** Sensorimotor processing, Animal behaviour

## Abstract

We have investigated the paths taken by Budgerigars while flying in a tunnel. The flight trajectories of nine Budgerigars (*Melopsittacus undulatus*) were reconstructed in 3D from high speed stereo videography of their flights in an obstacle-free tunnel. Individual birds displayed highly idiosyncratic flight trajectories that were consistent from flight to flight over the course of several months. We then investigated the robustness of each bird’s trajectory by interposing a disk-shaped obstacle in its preferred flight path. We found that each bird continued to fly along its preferred trajectory up to a point very close to the obstacle before veering over the obstacle rapidly, making a minimal deviation to avoid a collision, and subsequently returning to its original path. Thus, Budgerigars show a high propensity to stick to their individual, preferred flight paths even when confronted with a clearly visible obstacle, and do not adopt a substantially different, unobstructed route. The robust preference for idiosyncratic flight paths, and the tendency to pass obstacles by flying above them, provide new insights into the strategies that underpin obstacle avoidance in birds. We believe that this is the first carefully controlled study of the behaviour of birds in response to a newly introduced obstacle in their flight path. The insights from the study could also have implications for conservation efforts to mitigate collisions of birds with man-made obstacles.

## Introduction

Recently, there has been growing interest in understanding how birds cope with the challenges of short-range navigation and guidance. The motivation for these studies has been to understand the principles of visually guided flight in airborne creatures, as well as to enquire whether some of these biologically evolved principles can be applied to the design of algorithms for the guidance of unmanned aerial vehicles. Some aspects of visually-guided flight in birds are now beginning to be investigated – such as flight through narrow tunnels^1,2^, regulation of flight speed^3^, flight between obstacles^4,5^, collision avoidance^6,7^ choice of landing locations^8^, flight through narrow apertures^9^, and body awareness^10^. However, this challenging area of research is still in its infancy. Much still remains to be learned about the visual cues that are used to guide various manoeuvres, whether the cues used depend upon the context, and whether they are used in the same way across all bird species.

In this paper we investigate the paths taken by individual birds when they fly in a 25 m tunnel, which is long enough to encourage flight speeds that approach those in natural outdoor flight. We investigate the following questions: First, do all birds use the same flight trajectory, or does the preferred trajectory vary from bird to bird? Second, if the birds exhibit individually different flight paths, does this idiosyncratic preference persist with the passage of time? Third, if an obstacle is placed in a bird’s preferred path, does it switch to an entirely different flight path, or try to retain its originally preferred path by making just a short detour around the obstacle? The answers should not only provide insights into obstacle avoidance in bird flight, but may also have implications for the siting of new structures such as wind turbines and buildings.

## Materials and Methods

### Ethics statement

All experiments were carried out in accordance with protocols approved by the Australian Law on the protection and welfare of laboratory animals, and also by the Animal Experimentation Ethics Committees of the University of Queensland, Brisbane, Australia.

### Subjects

English adult Budgerigars, *Melopsittacus undulatus* – four birds, approximately 6–8 years old [*Drongo, Four, Nemo*, and *Two*] – together with five wild-type adult Budgerigars, approximately 2–3 years old [*Halley, Antares, Algol, Pluto*, and *Keppler*] served as subjects for the experiments. The average weight of the birds was 44.69 g, the average left wing and right wingspans (measured from the base of the wing to the wingtip) were each 13.09 cm, and the average body width was 3.96 cm (see Table S1 for details). The birds were purchased from various local breeders at the age of approximately one month and were housed in a communal mesh walled semi-outdoor aviary at The University of Queensland’s Pinjarra Hills field station. The aviary measured 4 m in length, 2 m in width and 2.2 m in height, and provided a natural diurnal light cycle. The birds also had access, through a window, to a climate-controlled indoor room (length 7.63 m, width 2.03 m and height 2.31 m) in an adjoining building, allowing protection from inclement weather. When participating in flight trials (about two to three times a week, only when the recordings for this project were conducted), the birds were moved to an experimental flight tunnel located at the same station. During the experiments birds were kept in the tunnel in groups of up to four in small cages (47 $\times$ 34.5 $\times$ 82 cm) for a duration of not more than seven hours per day. After the completion of each day’s experiment, the birds were returned to the aviary.

### Experimental configuration

The experiments took place indoors in a tunnel 25 m long, 1.4 m wide and 2.50 m high, with white side walls, a grey floor and a meshed ceiling. Both ends of the tunnel were covered with a white curtain, to enhance the visual contrast of the bird and facilitate its detection and tracking in the video images (see details below).

**Figure 1:**
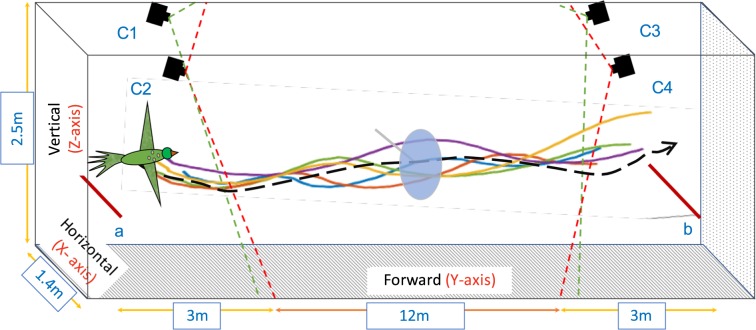
Experimental setup. Birds were trained to fly from point a to b, and from b to a. The mean preferred path for each bird (dashed black line) was calculated by analysing 5 flights (coloured lines). An obstacle (a blue disk, diameter 41cm) was then placed midway along the length of the tunnel to obstruct the preferred path of each bird, and the flights were re-filmed. The flights were recorded using four synchronised high-speed cameras (C1–C4), mounted on the side walls. The figure is not to scale. Further details in ‘Materials and Methods’.

Each bird was initially trained to take off from a perch at one end of the tunnel (*a*, Fig. 1), fly through the tunnel (with or without an obstacle, depending upon the experiment), and land on a bird cage (1.2 m high) at the other end of the tunnel (*b*, Fig. 1). Five such flights were conducted, before video recording of their flights was commenced. The take-off perch was hand-held by an experimenter, and a slow rotation of the perch induced the bird to take off.

Two scenarios were investigated: (a) an *obstacle-free scenario*, in which the birds were filmed as they flew in the empty, obstacle-free tunnel; (b) an* obstacle scenario*, in which the birds’ flights were filmed when an obstacle was placed in the tunnel. Two rounds of experiments were conducted in the obstacle-free scenario. *Round 1* served as a baseline for establishing each bird’s preferred flight path in the empty tunnel. *Round 2* was conducted eight months later, to check whether the birds retained their originally preferred path. During the intervening interval of 8 months, the birds participated in other experiments, outside the scope of this study, that used a different experimental configuration. As will become evident in the Results section, individual birds displayed consistent, but idiosyncratic flight paths during flight in the obstacle-free scenario.

The obstacle scenario experiments were commenced 4 days after the completion of the Round 2 of the obstacle-free scenario. In the obstacle scenario each bird was confronted with an obstacle, placed midway along the tunnel. The obstacle consisted of a disk, 41 cm in diameter, made from thin blue cardboard, supported by a thin horizontal dowel (diameter 3 mm). The disk was placed in the X-Z plane at the midway point of the tunnel, i.e, halfway along the Y axis. The position of the disk in this median X-Z plane was determined separately for each bird and flight direction, to ensure that it would obstruct the flight path specific to each bird. This position was determined by evaluating, for each bird and flight direction, the mean position of the flight trajectories in the median X-Z plane, as measured over a 2 m flight segment spanning a range $\pm 1$ m from this plane in Round 2 of the obstacle-free scenario. The centre of the disk was placed at this position in the median X-Z plane, which was calculated separately for each bird and flight direction (a to b, or b to a). Due to the robustness of the trajectories of individual birds it turned out that, from the perspective of each bird, the position of the disk was very similar for the two flight directions. The diameter of the disk was chosen to be roughly comparable to the average variability from flight to flight in each bird’s position in the X-Z plane in Round 2 of the obstacle free scenario. Thus, the diameter of the disk, and its bird-specific positioning, ensured that the disk would very likely constitute an obstacle, for each bird, if the bird chose to retain the trajectory that it had flown in the obstacle-free scenario.

Nine birds (*Drongo, Four, Nemo, Two, Halley, Antares, Algol, Pluto*, and *Keppler*) were tested individually in Rounds 1 and 2 of the obstacle-free scenario, and in the obstacle scenario. For Round 1 of the obstacle-free flights, after the training phase we recorded 5 flights from each side of the tunnel for each bird, summing up to a total of 90 flights. For Round 2 of the obstacle-free flights, we recorded 2 flights from each side for each bird, summing up to a total of 36 flights. Finally, for the obstacle scenario, we recorded 5 flights from each side for each bird, summing up to a total of 90 flights. Altogether, 216 flights were recorded in the course of the study.

### Recording

The flights were recorded using a network of four synchronised Emergent HS-4000 ultra-high speed 10GigE 4-mega-pixel cameras (C1, C2, C3 and C4 in Fig. 1). The cameras were mounted on the two side walls at both ends of the tunnel at a height 2.2 m, as illustrated in Fig. 1. Each camera was equipped with a 12 mm focal length lens to maximise the image resolution within the region of interest. Each flight yielded four synchronised image sequences. Flights were recorded at 100 frames per second, which provided adequate temporal resolution for detecting and tracking the birds.

### Reconstruction of 3D trajectories

For the reconstruction of the birds’ flight trajectories in 3D, cameras C1 and C2 were used for flights from point b to a, and cameras C3 and C4 for flights from a to b (see Fig. 1). This selection of camera pairs enabled reliable detection of the bird from the take off point and ensured consistent tracking of the location of the bird’s head in each video frame, regardless of the flight direction. The videos were recorded at a resolution of 2040 $\times$ 1080 pixels. The videos of each flight from the two cameras were initially processed using a bird detection and tracking algorithm to locate the centroid of the bird’s head location in each frame, and the positions in stereo frames were then combined to generate the 3D trajectory of the bird’s flight. Details of the tracking algorithm are given in^11^ and^12^. Stereo calibration of the cameras was carried out using a reference checkerboard pattern (check size 82.5 mm) in association with the Stereo Camera Calibrator Toolbox for Matlab (MathWorks®). This procedure delivered the calibration parameters for each camera, and also determined the precise 3D position and orientation of one camera relative to the other. The mean overall reprojection error in our experimental configuration was estimated to be 0.19 pixels, as reported by the error estimator built into the Stereo Camera Calibrator Toolbox. This figure provides an estimate of the accuracy of the camera calibration parameters. Based on the pixel resolution and camera geometry, we estimate the 3D resolution of the camera system in the X, Z and Y directions to be (1.9 mm, 1.7 mm, and 5.2 mm) at the nearest filming distance (6 m ahead of the obstacle plane); (7.8 mm, 7.8 mm, and 98.4 mm) at the plane of the obstacle; and (10.9 mm, 10.9 mm, and 191.0 mm) and at the farthest filming distance (3 m beyond the obstacle plane). Although the 3D resolution deteriorates with increasing distance from the camera, especially in the Y direction (as would be expected from the camera geometry), we do not believe that this compromises the positional estimates significantly because these are estimates of errors in individual frames, which are averaged out by smoothing (median-filtering) the position estimates over 5 frames; and the pixellation errors are statistically independent across frames.

We computed the 3D position and flight speed profiles (Fig. S1) of the birds, and plotted the variation of the radial distance of the bird with respect to the obstacle, across each flight. We also computed the profile of the optic flow (speed of image motion) generated by the obstacle in the visual system of the experimental bird during its flight through the tunnel. This calculation is illustrated in Fig. S2. These profiles are plotted as a function of the axial position in the tunnel (position along the Y axis, see Fig. 1), and were computed for the entire distance over which the flights were filmed (from 6 m ahead of the obstacle to 3 m behind it).

### Statistical evaluation

In order to test whether individual birds followed preferred flight paths, we performed a nearest-neighbour evaluation for each set of flights and compared each set to a set comprising an equal number of flights from randomly selected individuals, using the Wilcoxon signed rank test for paired samples. This was done using R with the arttool and emmeans packages^13^. For further statistical analysis of the general flight profiles, for example comparing the horizontal and vertical positions of the birds at the point of crossing the obstacle across the rounds and scenarios, we used the Aligned Rank Transformed (ART) ANOVA test using a linear mixed effects model^13^, with the Experimental Condition and Direction of Release as fixed effects and the Bird as a random effect. This type of ANOVA does not require the data to be normally distributed; we did, however, confirm that the variances of the samples were homogeneous. For post hoc comparison, we employed least squared means using the Tukey method for multiple comparisons. In order to determine if there was any relationship between radial distance and flight speed we employed a linear mixed effects model using the lme4 package in R.

In order to test whether the birds displayed a preference for passing the obstacle (disk) in a particular direction (for example, above or below it, or to its right or left), we used the methods of circular statistics. The direction in which a bird (bird $i$) cleared the disk was quantified by computing the mean direction over its 10 flights, and representing this mean direction by a unit vector ${\bar{r}}_{i}$. The mean clearing direction, $\bar{R}$, averaged over the nine birds, was then computed as the average of the nine unit vectors (one per bird)^14^: $\bar{R}=(\frac{1}{9}){\sum }_{i=1}^{9}{\bar{r}}_{i}$

The direction of $$\bar{R}$$ indicates the overall mean clearing direction (averaged over all 9 birds), and the length of $$\bar{R}$$, $$(| R| )$$, provides a measure of the coherence in the clearing direction across the nine birds: $$| R| $$ has a maximum value of 1 when all birds display exactly the same clearing direction, and a minimum value of 0 when the clearing directions are distributed randomly^14^. The Rayleigh test^14^ was used to obtain a $$P$$ value for ascertaining whether the distribution of clearing directions, across all birds, was significantly different from random.

## Results

### Obstacle-free flights

**Figure 2:**
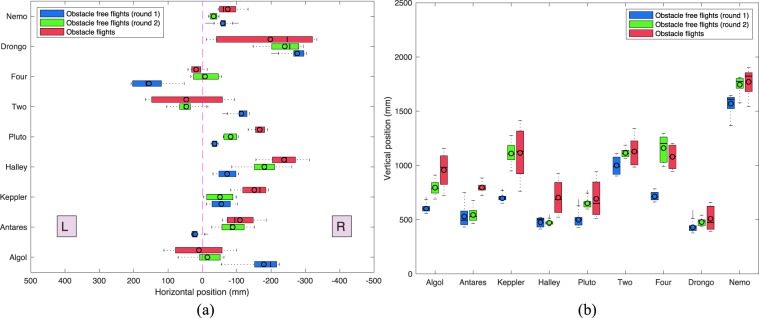
Box-and-whisker plot showing the average relative horizontal position (**a**) and altitude (**b**) of each bird - averaged over the entire flight - for Rounds 1 and 2 of the obstacle-free scenario, and the obstacle scenario. The black circles represent the mean values and the black dashes represent the median values. The box represents the middle 50 percent of values for the group. The upper and lower whiskers represent values outside the middle $50 \%$. Data not included between the whiskers, i.e. outliers, are represented as grey lines. In (**a**) the pink dashed line depicts the midline of the tunnel. R and L denote positions to the right and left of the tunnel, respectively, from the bird’s point of view.

In Round 1 of the obstacle-free scenario, we noticed a high propensity for individual birds to adhere to specific flight paths. This is illustrated in Fig. 2, which shows the mean horizontal position (Fig. 2a) and the mean vertical position (Fig. 2b) for each bird, averaged over its entire flight. To verify the tendency of each bird to fly along a specific trajectory, we used a nearest neighbour approach to compare the average nearest-neighbour distance between the 10 flights of each bird, with the nearest-neighbour distance between flights from randomly selected birds. The nearest-neighbour distance was calculated along each point of the flight trajectory, and not at a specific distance along the length of the tunnel. We found that the set of flights for each bird had a significantly smaller nearest-neighbour distance, compared to the nearest-neighbour distance for flights of different birds, selected pairwise, randomly across the population (row 1, Table 1). A nearest-neighbour analysis of the flights in Round 2 yielded the same result (row 2, Table 1). This indicates that each bird indeed tended to adhere to its preferred flight trajectory.

**Table 1:** Grand median of the average nearest-neighbour distance (mm) for each set of flights and the respective randomised set of flights.

	Grand median of nearest-neighbour distances for intra-individual flights	Grand median of nearest-neighbour distances for randomly selected flights	Wilcoxon signed rank test
Obstacle-free flights (Round 1)	233	436	$V=0;p=7.629{e}^{-06}$
Obstacle-free flights (Round 2)	212	471	$V=22;p=0.004$
Obstacle flights	236	513	$V=0;p=7.629{e}^{-06}$

The tendency of each bird to fly a fixed path was evident for flights in both directions in the obstacle-free scenario. This is illustrated in Fig. S3, which shows the mean and standard deviation of the position of each bird in the cross section of the tunnel (from the bird’s viewpoint), during its flights in the forward and the reverse directions in Round 1. The mean X and Z positions of the flight trajectories were not significantly different for the flights in the two directions ($p > 0.46$) for X and Z; two-tailed t-test). This further confirms the robustness of each bird’s preferred path.

In Round 2 of the obstacle-free scenario, there was again a strong tendency for individual birds to adhere to specific flight paths (see Fig. 2a,b). Again, each set of flights for a given bird had a significantly smaller nearest-neighbour distance compared to a set of flights selected randomly across the population (see row 2, Table 1), indicating that each bird maintained a robust flight path.

A comparison of the results obtained for Rounds 1 and 2 of the obstacle-free scenario reveals that, in both cases, there is a tendency for the birds to fly slightly to their right of the mid-line of the tunnel (irrespective of the flight direction). However, for the most part (with three exceptions, see below), there is no major change in the overall mean horizontal position or the overall mean vertical position of each bird’s preferred trajectory between Rounds 1 and 2. This is confirmed in the plot of Fig. S4a, which compares the mean positions of each bird’s trajectory in the cross section of the tunnel, across the two rounds. While three birds (*Algol*, *Four* and *Keppler*) changed their mean positions between the two rounds – mostly in the vertical plane – the other six birds maintained approximately the same average positions (see Fig. S4a, squares vs circles). The difference between the mean X positions, averaged over all birds, is 9.76 $\pm$ 6.70 cm, and the difference between the mean Y positions, averaged over all birds, is 17.14 $\pm$ 16.32 cm.

**Figure 3:**
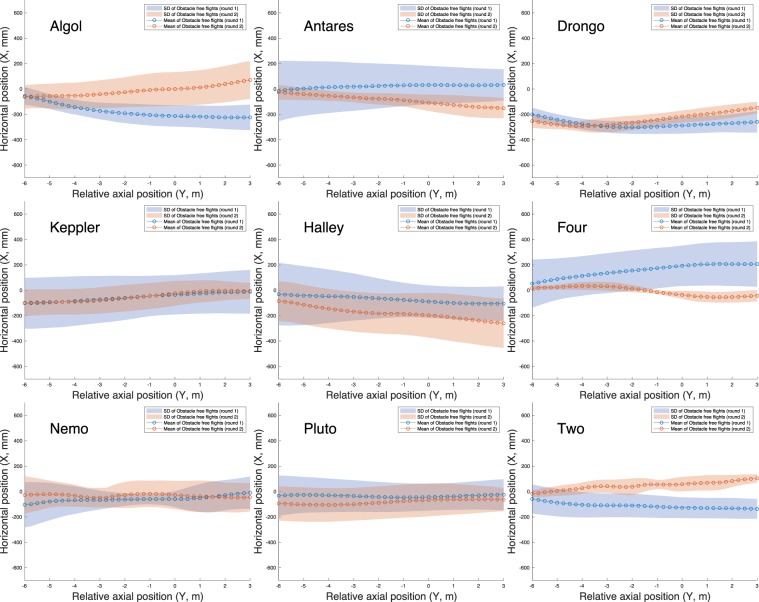
Horizontal positions of each bird’s flight path (in a top view), with mean and standard deviation. 9 birds, 10 flights per bird per experimental scenario.

**Figure 4:**
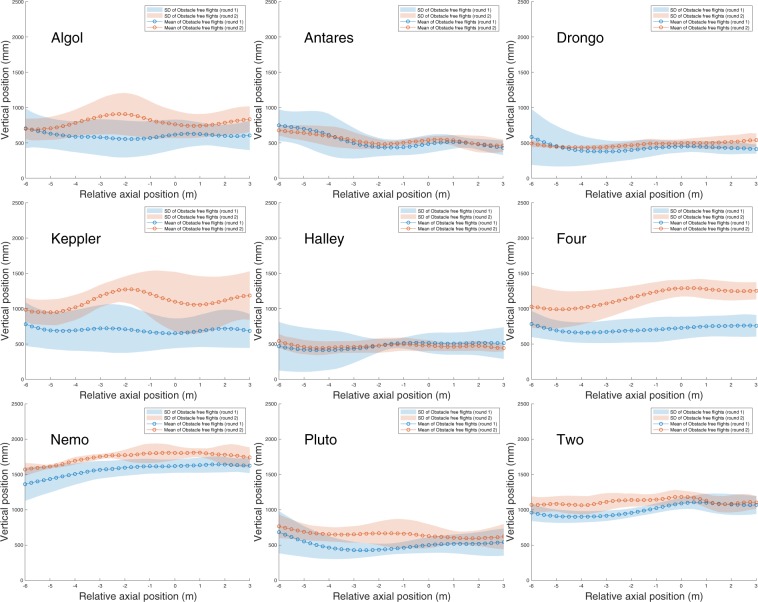
Vertical positions of each bird’s flight path (in a side view), with mean and standard deviation. 9 birds, 10 flights per bird per experimental scenario.

Thus, the majority of the birds maintained their preferred trajectories over the 8-month interval between the experiments conducted in Rounds 1 and 2 of the obstacle-free scenario. Figures 3 and 4 further demonstrate that individual birds not only maintain their individual mean positions in the cross section of the tunnel, but also retain their preferred paths, displaying a small standard deviation. A detailed statistical comparison of these results is given later below, in the section ‘Statistical Analysis of Flight Trajectories’.

### Obstacle flights

**Figure 5:**
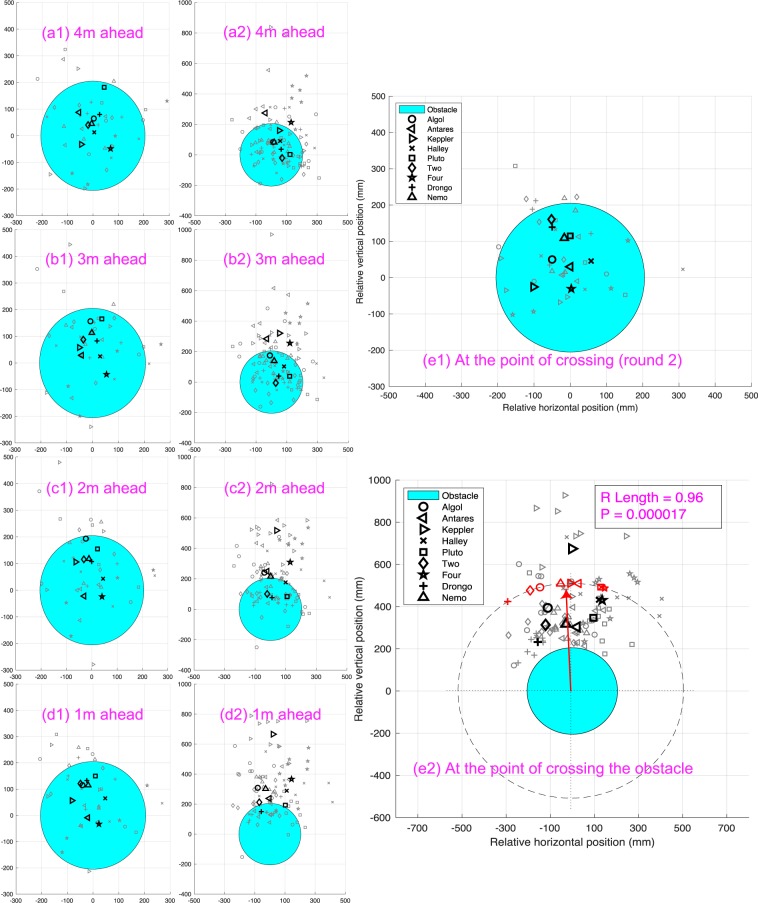
Position of each bird in the cross section of the tunnel for each of its 4 flights in Round 2 of the obstacle-free scenario (small symbols), relative to the position at which the disk (blue circle) was placed in the subsequent tests in the obstacle scenario, at different axial distances from the disk, as well as at the point of crossing. Panels (a1–e1) pertain to the obstacle-free scenario and panels (a2–e2) pertain to the obstacle scenario. The large symbols represent the mean position of each bird, averaged over 4 flights. Table S4 provides the mean and the SD of the angular position at which each bird, shown here in Fig. e2, passes the disk.

The question then arises: How does a bird behave if an obstacle is interposed in its preferred flight path? To examine this, we recorded 10 flights of each bird when an obstacle, consisting of a 41 cm diameter disk, was positioned halfway along the tunnel with its centre located at the mean (X,Z) position of each bird’s trajectories in Round 2 of the obstacle-free scenario, as described in ‘Materials and Methods’. Figure 5 – a1,b1,c1,d1, and e1 shows the position of each bird in the cross section of the tunnel in Round 2 of the obstacle-free scenario, relative to the position at which the disk was placed in the subsequent tests in the obstacle scenario. It is clear that the mean position of each bird lies within the projected cross section of the disk, at distances of up to 4 m ahead of the position at which the disk was placed in the subsequent obstacle tests. Thus, the disk was squarely in the flight path of each bird, as measured in Round 2 of the obstacle-free scenario.

The obstacle tests were commenced 4 days after the tests in Round 2 of the obstacle-free scenario had been completed. Hence, it was very unlikely that the birds would have changed their flight path preferences substantially during this short interval.

Figure S4b compares the mean positions of each bird in the obstacle scenario and in Round 2 of the obstacle-free scenario, over a 2 m flight segment spanning the position of the obstacle ($\pm 1$ m from the obstacle). The dashed circles indicate the size and position of the obstacle (disk) for each bird. It is evident that each bird avoids the disk by flying above it.

The avoidance manoeuvre is analysed in greater detail in Fig. 5(a2–e2), which shows scatter-plots of the radial positions of the birds relative to the obstacle when they were at distances of 4 m, 3 m, 2 m, and 1 m ahead of the obstacle, and at the point of crossing the obstacle. Even in the presence of the obstacle, the birds maintained their preferred paths up to a point 3 m ahead of the obstacle (Fig. 5a2), beyond which they started to veer away progressively (Fig. 5b2,c2,d2), eventually clearing the obstacle by flying above it (Fig. 5e2). The veering response to avoid the obstacle is quantified by the magnitude and direction of the mean vector $\bar{R}$ (Fig. 5e2), computed as described in ‘Materials and Methods’. $\bar{R}$ is directed upwards, has a magnitude close to 1.0, and is highly significant ($P < 0.00002$), indicating that the obstacle is avoided by flying above it.

Inspection of the videos (90 in all, one for each flight) shows no evidence of speed reduction, outstretched legs, or change in the pattern or phase of the wingbeat as the birds approach the obstacle. The velocity profiles (Fig. S1) indicate that the birds do not slow down while approaching the obstacle. Thus, there is no evidence of the birds attempting to land on the obstacle when they encounter it.

Figure S5 examines the obstacle avoidance response in another way, by charting the percentage of flights that are on a collision course with the obstacle, as a function of the distance from the obstacle. This is done by determining, at each distance, the number of flights in which the bird is positioned within the volume of the axial cylinder projected by the disk (red columns), and outside it (grey columns), as can be visualised in Fig. 5. Figure S5 indicates a substantial decrease in the percentage of collision-directed flights between 3 m and 2 m, suggesting that collision avoidance commences at a distance of $\sim 2.5$ m from the obstacle. The blue/green columns in Fig. S5 show the corresponding numbers for Round 2 of the obstacle-free scenario, for a ‘virtual’ disk placed at the position where it was located in the obstacle tests. In this case the percentage of flights within the virtual disk does not change substantially – it is more or less constant at a high level $(70-75 \% )$, demonstrating that, in the obstacle tests, the disk was indeed placed directly in each bird’s preferred flight path, as measured in Round 2 of the obstacle-free scenario.

Finally, a nearest-neighbour analysis of the obstacle flights reveals that, as in the obstacle-free scenario (Rounds 1 and 2), each set of flights for a given bird in the obstacle scenario has a significantly smaller nearest-neighbour distance compared to a set of flights selected randomly across the population (see row 3, Table 1), indicating that each bird tends to largely retain its preferred flight path when avoiding the obstacle.

### Control of radial separation from the obstacle

**Figure 6:**
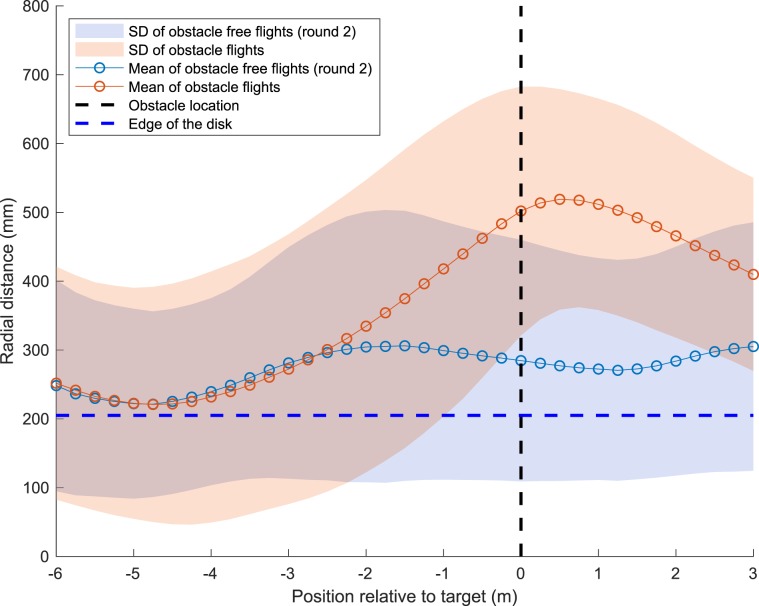
Mean radial distance profiles and standard deviation (SD) for the flights in Round 2 of the obstacle-free scenario, and the obstacle scenario. The vertical dashed line depicts the position of the disk. The horizontal dashed line indicates the boundary of the disk.

Next, we investigated in greater detail how the birds controlled their radial distance from the obstacle (disk) during their flight. The radial distance is the defined as the distance in the X-Z plane from the bird’s head to the centre of the disk, when the disk was present. Figure 6 shows the profiles of the mean radial distance for the obstacle scenario (orange circles), and for Round 2 of the obstacle-free scenario (blue circles). The two profiles are similar up to a point 3 m ahead of the obstacle. At distances closer to the disk, the birds begin to veer away from the disk when it is present, achieving a maximum radial separation of about 500 mm slightly beyond the point of crossing the disk. This radial separation represents a distance of 295 mm from the edge of the disk, which has a radius of 205 mm.

Figure S6 shows the difference between the mean radial distance profiles for Round 2 of the obstacle-free scenario, and the obstacle scenario shown in Fig. 6, normalised to a value of 100. During the initial phase ($-6$ m to $-2$ m, highlighted by the yellow rectangle) the difference between the two profiles is less than 10%, indicating that, during the initial part of their flight, the birds maintain their preferred trajectories regardless of the presence or absence of an obstacle in their path. In the presence of an obstacle, the birds begin to deviate from their preferred trajectory only when they are about 2.5 m away from the obstacle. After passing the obstacle the birds tend to return to the originally preferred obstacle-free trajectories, as measured in Round 2 of the obstacle-free scenario.

**Figure 7:**
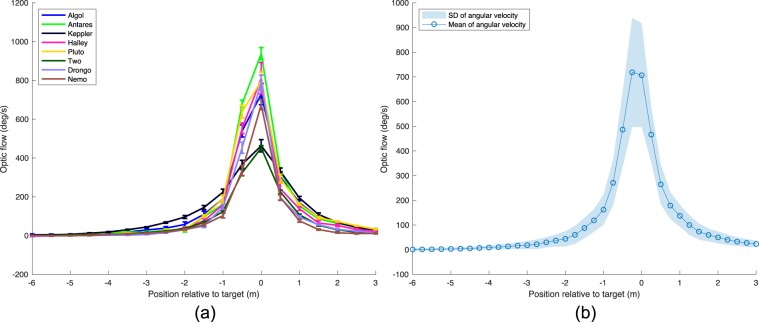
Profiles of optic flow (image angular velocity, in deg/s) generated by the the obstacle at each point of the flight. (**a**) Average profiles for individual birds, showing mean values and standard errors of the means (SEMs); (**b**) Mean profile and standard deviation, averaged over all birds.

We also computed, for the obstacle scenario, the time course of the optic flow (image angular velocity) generated by the disk as the bird flew past it (Fig. 7a,b). The procedure for calculating the optic flow is illustrated in Fig. S2. The magnitude of the optic flow increases rather sharply as the bird approaches the disk, reaching a maximum close to the point of crossing the disk. The mean magnitude of the maximum optic flow, averaged over all birds, is about 700 deg/s (Fig. 7b). However, the maximum value of the optic flow is not the same across all the birds: It is clear from Fig. 7a that Antares and Halley experience significantly higher levels of optic flow than does Nemo; and all three of these birds experience significantly higher levels of optic flow than do Keppler and Two. Furthermore, there is no systematic relationship between these maximum optic flow levels and the average flight speeds of the individual birds (see Table S2). This raises the interesting possibility that different birds have different ’set points’ for the maximum allowable optic flow.

This raises the possibility that, while flying past the disk, the birds are maintaining a safe distance from the the disk by ensuring that the mean magnitude of the peak optic flow generated by the disk does not exceed 700 deg/s.

If the birds are sensing the optic flow generated by the the disk and using it to control the radial distance to the disk, they would need to hold their flight speed constant – only then can the optical flow be directly calibrated in terms of distance. Table S2 compares the coefficients of variation (CV) of the flight speed, radial separation, radial proximity [$=\frac{1}{radialseparation}$], and optic flow. The coefficient of variation is a well-accepted measure for comparing the relevance of various candidate visual cues in controlling a particular behaviour [e.g.^15^]. It is clear from this table that the flight speed is the variable that has the lowest mean CV (0.08), which is significantly lower than the mean CVs of the optic flow (0.19) (Wilcoxon signed rank test: V = 1; p = 0.007812), the radial separation (0.24) (V = 0; p = 0.003906) and the radial proximity (0.25) (V = 0; p = 0.003906). This indicates that the speed of flight is tightly controlled, as one would expect if the birds were using the magnitude of the optic flow as a cue to gauge the radial distance to the obstacle. Although there is considerable variation in the mean flight speed across different individuals, each bird regulates its flight speed tightly, as is evident from the low standard deviations and low coefficients of variation shown in Table S2. This further highlights the robustness of each bird’s flight characteristics.

The mean CV of the optic flow is significantly lower than the mean CV of the radial separation (V = 4; p = 0.02734) and the mean CV of the radial proximity (V = 44; p = 0.007812). This suggests that the birds are controlling the magnitude of the optic flow more tightly than the radial separation or the proximity (which, in effect, is another measure of the radial separation). There is no significant difference between the CVs of the radial separation and the proximity (V = 35.5; p = 0.1376). Thus, optic flow is likely to be an important cue that is used by the birds to control their distance to the obstacle, although we cannot exclude the use of additional cues.

Figure S7 shows the mean %CV of the flight speed along the axis of the tunnel at various axial distances from the obstacle. These values are uniformly low, the largest value being 12.2%. Interestingly, the CV decreases as the bird approaches the obstacle and attains it lowest value in the vicinity of the obstacle, indicating tightest control of flight speed in that region. This observation, again, favours the optic flow hypothesis.

When the birds pass the obstacle, the magnitude of the optic flow (O) that they experience (in radians/sec) is equal to the ratio between the flight speed (S, in metres/sec) and the radial distance to the obstacle (R, in metres). This magnitude can also be expressed as the product of the flight speed and the proximity of the object (P, in units of $metre{s}^{-1}$), where $P=\frac{1}{R}$. Thus, $O=S\times P$. If the birds were attempting to hold the magnitude of the optic flow constant as they pass the obstacle, one would expect the radial distance to be larger (and the proximity to be lower) for higher flight speeds.

As a first step toward investigating this ‘optic flow’ hypothesis, we reconstructed a linear-mixed model to assess the overall relationship between speed and radial distance, with the bird as a random effect. This analysis revealed no statistically significant relationship between speed and radial distance (${\chi }^{2}=2.4443;p=0.118$) A similar linear-mixed model also revealed no significant relationship between speed and proximity (${\chi }^{2}=0.0229;p=0.8797$).

Another way to investigate the optic flow hypothesis is to use the relationship $O=S\times P$ to compute the covariance between the flight speed (S) and the proximity (P). If the birds are holding the optic flow constant, one would expect the proximity to be lower for higher flight speeds, and vice versa. In other words, the covariance between the flight speed (S) and the proximity (P) would be negative. A covariance of zero, on the other hand, would imply that flight speed and proximity are unrelated, i.e. statistically independent. The covariance between two random variables (in our case, S and P) can be calculated from the difference between the mean value of their product ($\bar{O}$) and the product of their individual mean values ($\bar{S}$ and $\bar{P}$)^16^. We have used this relationship to calculate the covariance between the flight speed and the proximity. The covariance is negative for all of the nine birds, as well as for the grand mean (see Table S3). This finding provides support for the optic flow hypothesis.

**Figure 8:**
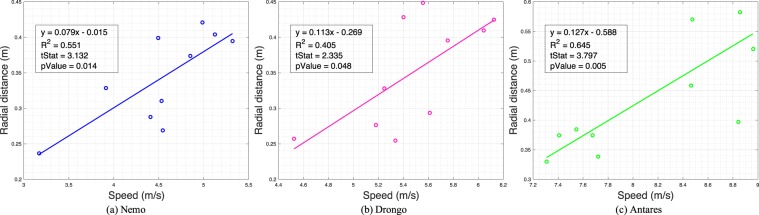
Examples of correlation between flight speed and radial distance to the obstacle at the point of crossing the obstacle, for those birds where the correlation is significant $(p < 0.05)$.

A third way to investigate the optic flow hypothesis is to examine the correlation between (i) flight speed and radial distance; and (ii) flight speed and proximity. Six of the nine birds showed no significant correlation between flight speed and radial distance; however, three of them (*Nemo*, *Drongo* and *Antares*) showed a significant positive correlation (p<0.05), implying that the radial distance increased with flight speed. The regressions for these three birds are shown in Fig. 8. The analysis of proximity revealed a very similar pattern. Again, six of the nine birds (the same six individuals) showed no significant correlation between flight speed and proximity; whereas three of them (the same three individuals) showed a significant negative correlation (p < 0.05, Fig. S8), implying that the proximity decreased with flight speed. Taken together, the data in Figs. 7, 8, S6 and S8 suggest that the birds are keeping a safe distance from the obstacle by ensuring that the maximum magnitude of the optic flow generated by the disk during the passage does not exceed a prescribed value. This value varies from bird to bird, and its mean value is in the vicinity of 700 deg/sec.

### Recovery after passing the obstacle

Figure S6 shows a steep reduction of the radial separation after the birds have passed the obstacle. Although the visual field of the stereo camera system did not extend beyond a distance of 3 m past the obstacle, the radial separation has already dropped to about 40% of its maximum value at this point, and the continued steep downward trend in the radial separation profile strongly suggests that each bird tends to return to its originally preferred trajectory soon after passing the obstacle. This notion is supported by the data in Fig. S4a, which compares, for each bird, the mean positions of the flight in the cross section of the tunnel in Round 2 of the obstacle-free scenario (circles) and in the obstacle scenario (triangles), averaged over the entire flight. The two mean positions are close together, for each bird. The average separation is $11.76\pm 7.6$ cm (SD), and never exceeds $23.88$ cm. This small separation suggests that each bird does indeed merge into its originally preferred trajectory after crossing the obstacle, and does not execute a new flight path.

### Statistical analysis of flight trajectories

The flights recorded in Rounds 1 and 2 of the obstacle-free scenario and in the obstacle scenario were analysed using ART-ANOVA statistics to look for scenario-dependent effects on the mean horizontal positions and altitudes of the trajectories. The analysis revealed that there was no significant effect of scenario on the mean horizontal position (ART-ANOVA: ${F}_{2,16}=0.55605;p=0.58416$). However, there was a significant effect of scenario on the mean vertical position (ART-ANOVA: ${F}_{2,16}=5.2214;p=0.017968$). Post hoc comparison revealed that this effect was limited to a change in the mean flight altitude between Round 1 of the obstacle-free flights and the obstacle scenario (lsmeans: $df=16;t=3.210;p=0.0143$). There was no significant change in the mean flight altitude between Round 2 of the obstacle-free scenario and the obstacle scenario (lsmeans: $df=16;t=1.284;p=0.4239$). The difference between Rounds 1 and 2 of the obstacle-free scenario did not quite reach statistical significance (lsmeans: $df=16;t\,=\,$ − $1.926;p=0.1636$). In order to compare the obstacle free flights of Rounds 1 and 2 in greater detail, we performed a Monte Carlo simulation. We calculated the average nearest neighbour distance of all flights in round 1 to all flights in Round 2 for each individual and compared it to the average nearest neighbour distance between Round 1 and 2 of two random birds. In order to acquire statistically stable results this process was carried out 10 times, to acquire 10 random combinations of birds for comparison. The average nearest neighbour distance had a median value of 352 mm for flights of the same individual, and 436 mm for random combinations of individuals. The two median values were significantly different (Wilcoxon signed rank test: $W=1439400;p < 7.0\times 1{0}^{-09}$), suggesting that flights of the same individual were more similar between Round 1 and 2, compared to random combinations of birds.

### Videos

Six videos (S1–S6) showing examples of the flight trajectories are available in the SI.

## Discussion

We have shown that Budgerigars display a high propensity to adhere to individually preferred flight paths, as indicated by the significantly smaller nearest neighbour distance of each set of flights compared to a set of random flights. Further statistical analysis of the flight trajectories (see above) corroborates our findings that (a) Budgerigars display flight trajectories that are robust, but idiosyncratic; and (b) the introduction of an obstacle in a Budgerigar’s flight path does not alter its trajectory in a major way. The bird largely retains its original trajectory, except for a brief detour to avoid the obstacle.

While our experiments were set in an artificial environment, similar behaviours have been reported for free ranging birds^17,18^. Like our birds, pigeons, after several releases from the same location, develop a behaviour called route stereotypy, where a given individual retreads the path it has previously taken. This behaviour is generally associated with pigeons navigating to their goal using a simple strategy known as ‘piloting’, which involves moving from one familiar landmark to the next, or using landmarks in more complex ways to guide the flight trajectory. The difference in our experiment is that the observed stereotypical behaviour is likely embedded in an intrinsic motor pattern that is not associated with external landmarks – given that the environment presented to our birds was largely devoid of landmarks, the flights were short, and the goal was always in view.

This raises the question as to why the birds show this kind of stereotypical behaviour. It is important to note that the individual birds’ preferences were broadly consistent over small time scales, as well as larger time scales. However, some birds displayed small changes in their preferences between Rounds 1 and 2 of the obstacle-free scenario, which were separated by 8 months. The reason for these changes remains to be explored. One possibility is that the distribution of the birds’ positional preferences is related to their behaviour in a flock, and that these positions are determined by hierarchy, which could have undergone a change in the intervening period. This would be a topic for future investigation.

Our results (Figs. 2, S4 and 5(a2–e2)) reveal that Budgerigars tend to avoid a small obstacle by flying above it, rather than below it, or to either side. In this context, it is important (and interesting) to note that some of the birds (such as *Nemo, Two, Four* and *Keppler*) flew at a rather high altitude (see Fig. 2b), thus requiring the obstacle to be placed at large heights – close to the ceiling of the tunnel – to obstruct their flight paths. Despite this, and despite the fact that there was a large clear space under the obstacle, even these birds avoided the obstacle by flying above it, rather than below it. Thus, all of the 9 birds avoided the obstacle by flying above it when it was placed in their flight path, regardless of the height of the obstacle. It is unlikely that the dowel had a significant influence on the bird’s trajectories because (i) it would have been barely visible, given its narrow diameter (3mm); and (ii) most passages were above the disk.

Another important aspect is that, at the point of crossing the disk, the birds maintained a mean clearance (distance between the head and the disk boundary) of 30.74 cm (see Fig. 6), which is comparable to the average wingspan (30.13 cm; note that the wingspan is the sum of the left and right wingspans, each measured from the base of the wing to the tip, and the width of the body between the two wing bases). The mean clearance for the closest 25% of the flights (mean of the closest quartile) is 11.18 cm, which is smaller than half the wingspan. Furthermore, in 15 of the total of 90 flights, the clearance is less than half the wingspan (and the bird happens to have it wings folded while passing the obstacle). This tight clearance ensures avoidance of the obstacle with a minimal deviation from the preferred flight trajectory – which speaks volumes for the body awareness of Budgerigars. This feature of body awareness has also been demonstrated in earlier studies investigating flights of Budgerigars through narrow vertical apertures^9,10^.

The most surprising observation, however, is that Budgerigars show a strong tendency to retain their preferred flight path even when an obstacle is introduced in the path. The obstacle is avoided with a minimal and potentially dangerous last-minute manoeuvre. Our data, analysed in two different ways (see Figs. S5 and S6) infer that the avoidance manoeuvre commences at a distance of $\sim 2.5$ m. The average flight speed of about 6 m/s (as shown in Table S2) implies that the initiation of the avoidance occurs less than half a second before the obstacle is encountered.

It is very unlikely that the reason for the late avoidance response is due to a lack of visibility of the obstacle. When the bird begins to veer away from the disk at a distance of 2.5 m, the disk would subtend a visual angle of 9.4 deg, and present a high contrast against the white background at the end of the tunnel. Earlier experiments, investigating the ability of Budgerigars to find and land on high-contrast disks of a similar size, confirm that such objects are clearly visible to the birds^8^.

Bhagavatula *et al*.^5^ provided evidence that Budgerigars (*Melopsittacus undulatus*) navigate safely through the middle of a tunnel by balancing the magnitude of optic flow (the speed of image motion) experienced by the bird’s eyes. Our findings provide evidence to support the notion that Budgerigars may also use optic flow to guide their flight while avoiding a stationary obstacle. A safe distance from the obstacle could be achieved by ensuring that the peak magnitude of the optic flow generated by the disk does not exceed a prescribed value - which varies from bird to bird, and ranges from about 500–1000 deg/sec. It has been proposed that hummingbirds navigate through the middle of a tunnel by balancing the rates of image expansion generated by the two flanking walls^2^. We believe that it is unlikely that the Budgerigars in our experiments are relying solely on image expansion cues to guide the avoidance of the obstacle in our experimental configuration. This is because, unlike the studies of bird flight in obstacle - free tunnels (e.g.^2,5^), the obstacle in question here is not the wall of the tunnel -which is oriented parallel to the flight path - but a small, discrete, planar object (a disk) that is oriented perpendicular to the flight path, whose visual image varies in a complex way as the obstacle is approached and passed. Image expansion could be effective in preparing the bird to veer in the appropriate direction when the disk is far away. However, as the disk is approached, these expansion cues would become increasingly unreliable, and will disappear altogether when the bird is passing the disk (because the image of the disk would then be just a line, with no area). Thus, at times close to passing the disk, the translational optic flow cues would provide much more reliable information about the distance to the disk.

It is noteworthy that the individual birds display a remarkably constant speed throughout their flight (Fig. S1). This greatly simplifies the use of cues based on optic flow to compute range, as the distance to a stationary obstacle can then be directly calibrated in terms of the magnitude of the optic flow induced in the eye^19^.

These findings suggest that care may need to be taken while rearranging plants, perches or other objects inside aviaries, setting up scaffolding or cranes at building sites, selecting locations for erecting telecommunication towers, power lines and wind turbines - and, more generally, while planning any abrupt changes that are likely to cause poorly visible obstacles to be placed in the way of flight paths that are known to be used frequently by birds.

## Supplementary information

Supplementary Information.

Supplementary Information2

Supplementary Information3

Supplementary Information4

Supplementary Information5

Supplementary Information6

Supplementary Information7
