# Supplementary material for: Budgerigars adopt robust, but idiosyncratic flight paths
Source: Sci Rep. 2020 Feb 13;10:2535. doi: 10.1038/s41598-020-59013-3 (PMC7018814; doi:10.1038/s41598-020-59013-3)
Supplement: Supplementary file 7 — Supplementary Information7 [file 41598_2020_59013_MOESM7_ESM.pdf]

Budgerigars adopt robust, but idiosyncratic flight paths

Supplementary Information

Debajyoti Karmaker, Julia Groening, Michael Wilson,
Ingo Schiffner and Mandyam V. Srinivasan

November 14, 2019

- Supplementary Figures : Figures S1-S8
- Supplementary Tables: Tables S1-S4
- Supplementary Videos: Videos S1 - S6

1 Supplementary Figures

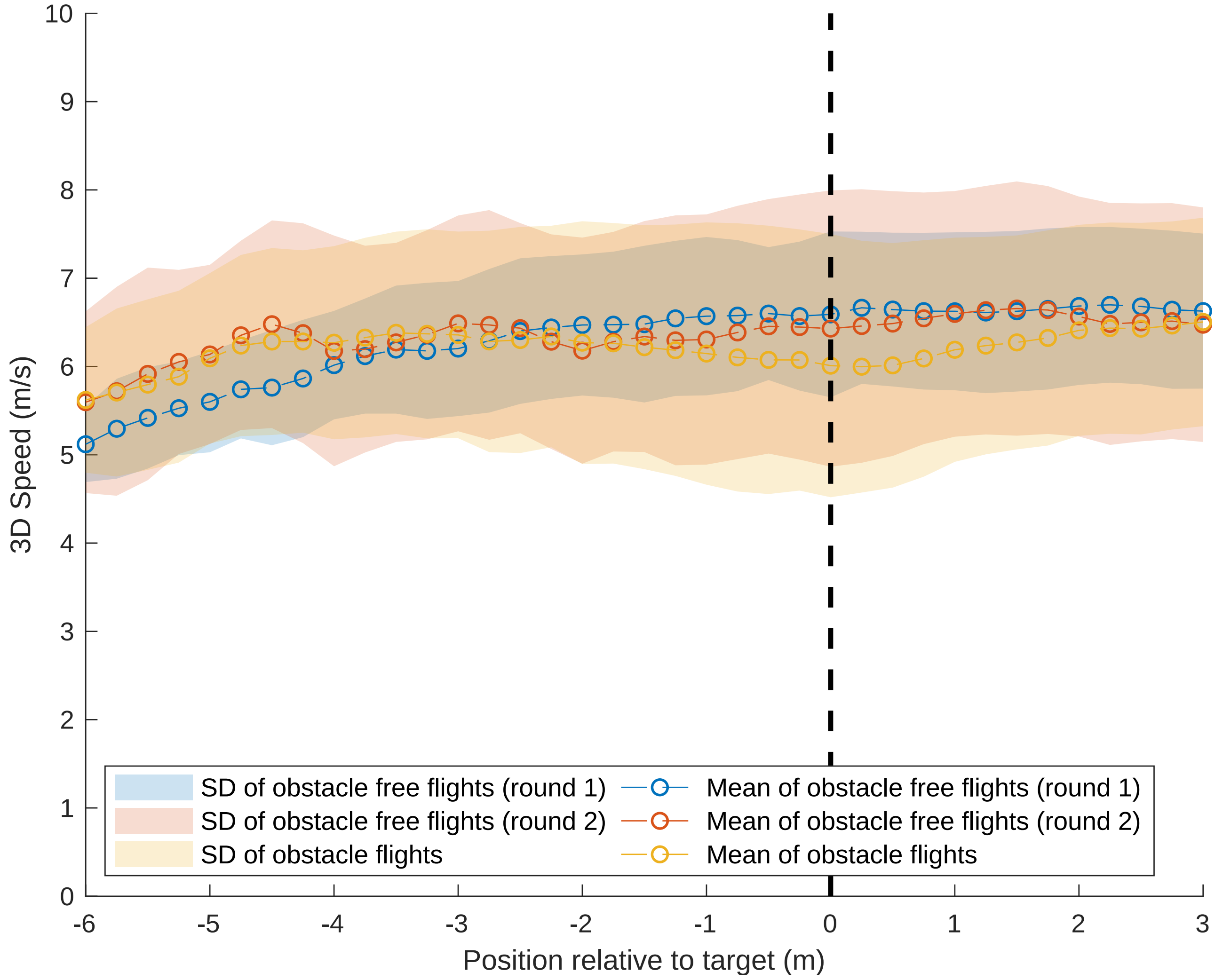

Figure S1: Profiles of 3D flight speed for Rounds 1 and 2 of the obstacle-free scenario, and the obstacle scenario. The vertical dashed line depicts the position of the disk.

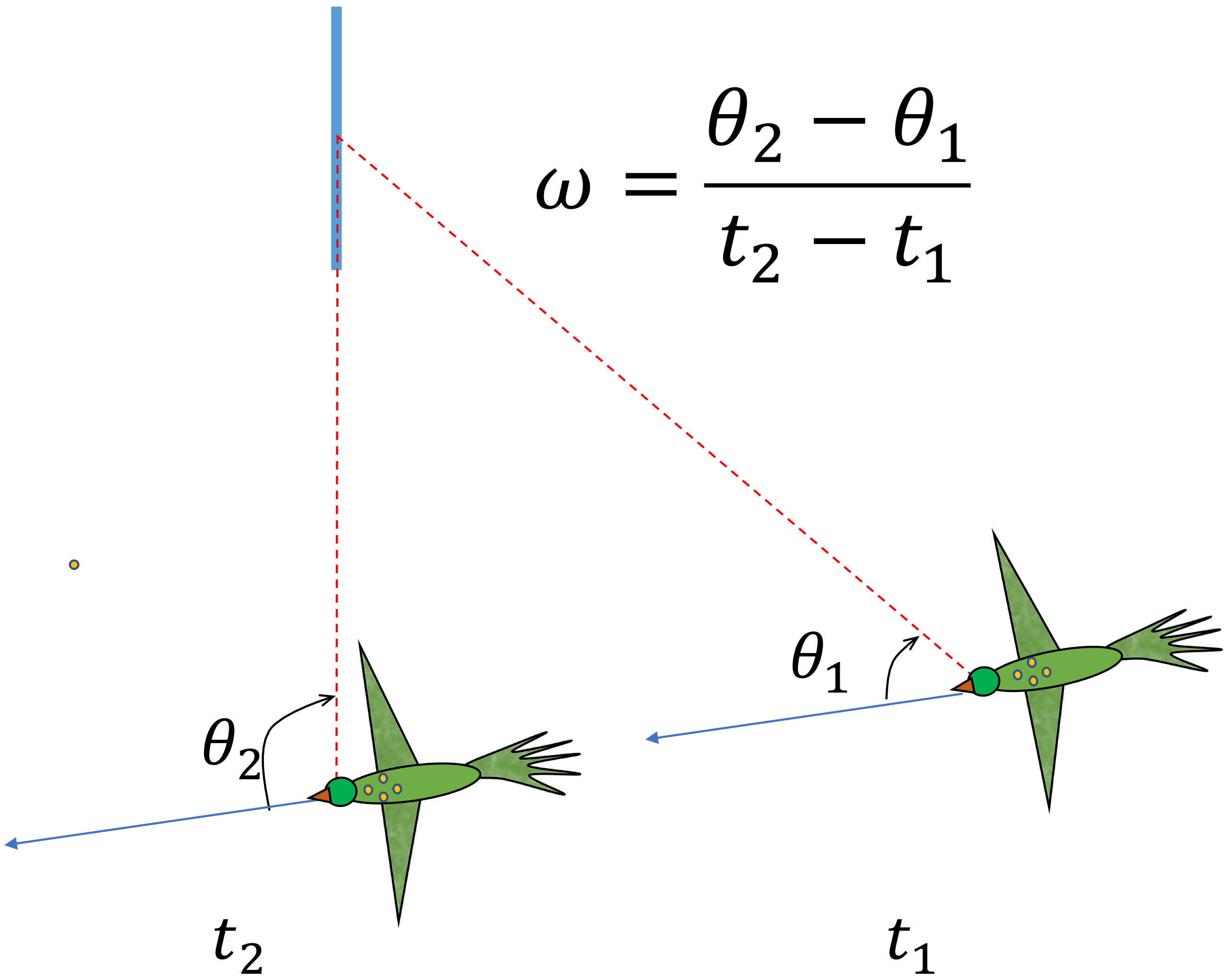

Figure S2: Computation of the instantaneous optic flow (ω, rad/sec) generated by the obstacle, from the rate of change of the bearing (θ) of the object.

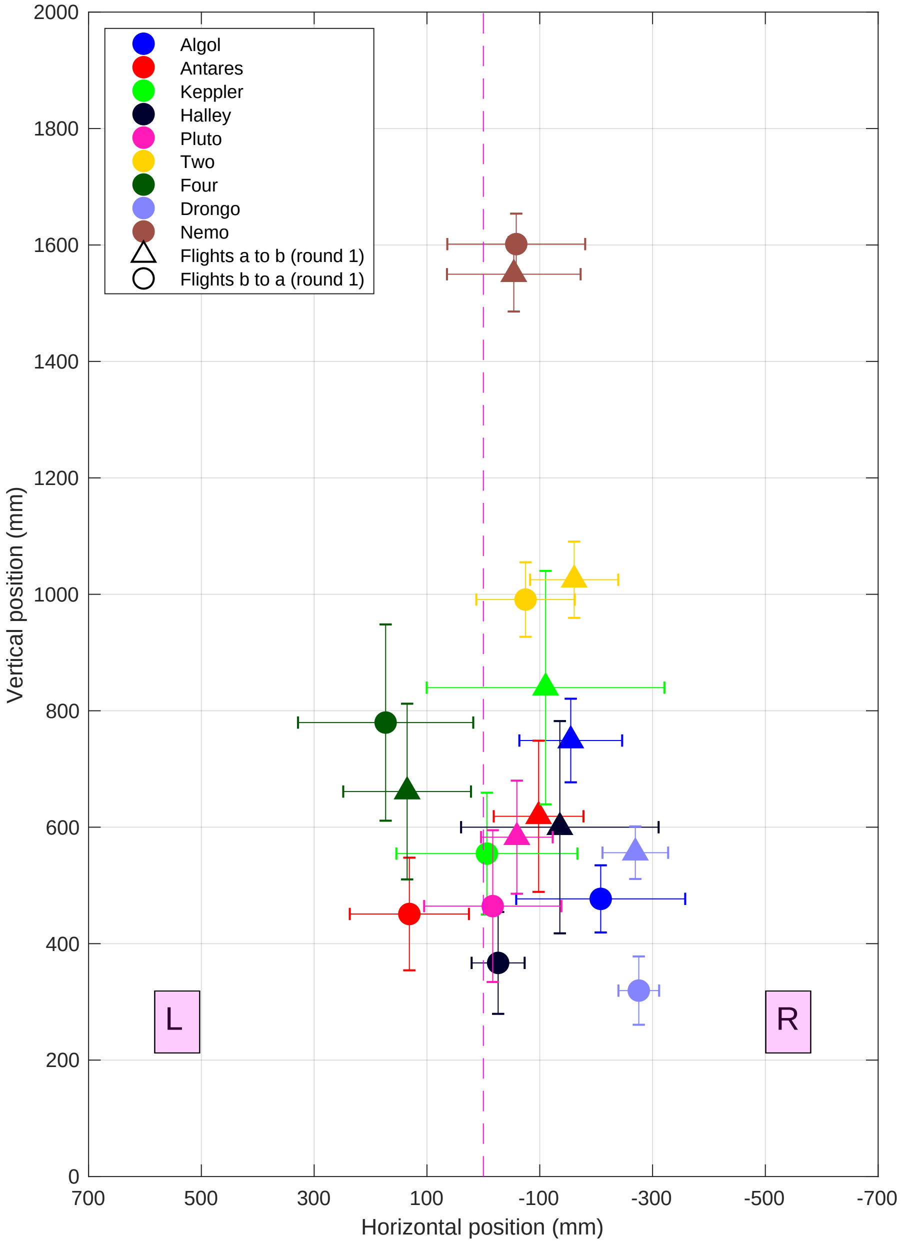

Figure S3: Mean and standard deviation of the position of each bird during its flights in the forward vs reverse directions in Round 1. R and L denote positions to the right and left of the tunnel, respectively, from the bird's point of view.

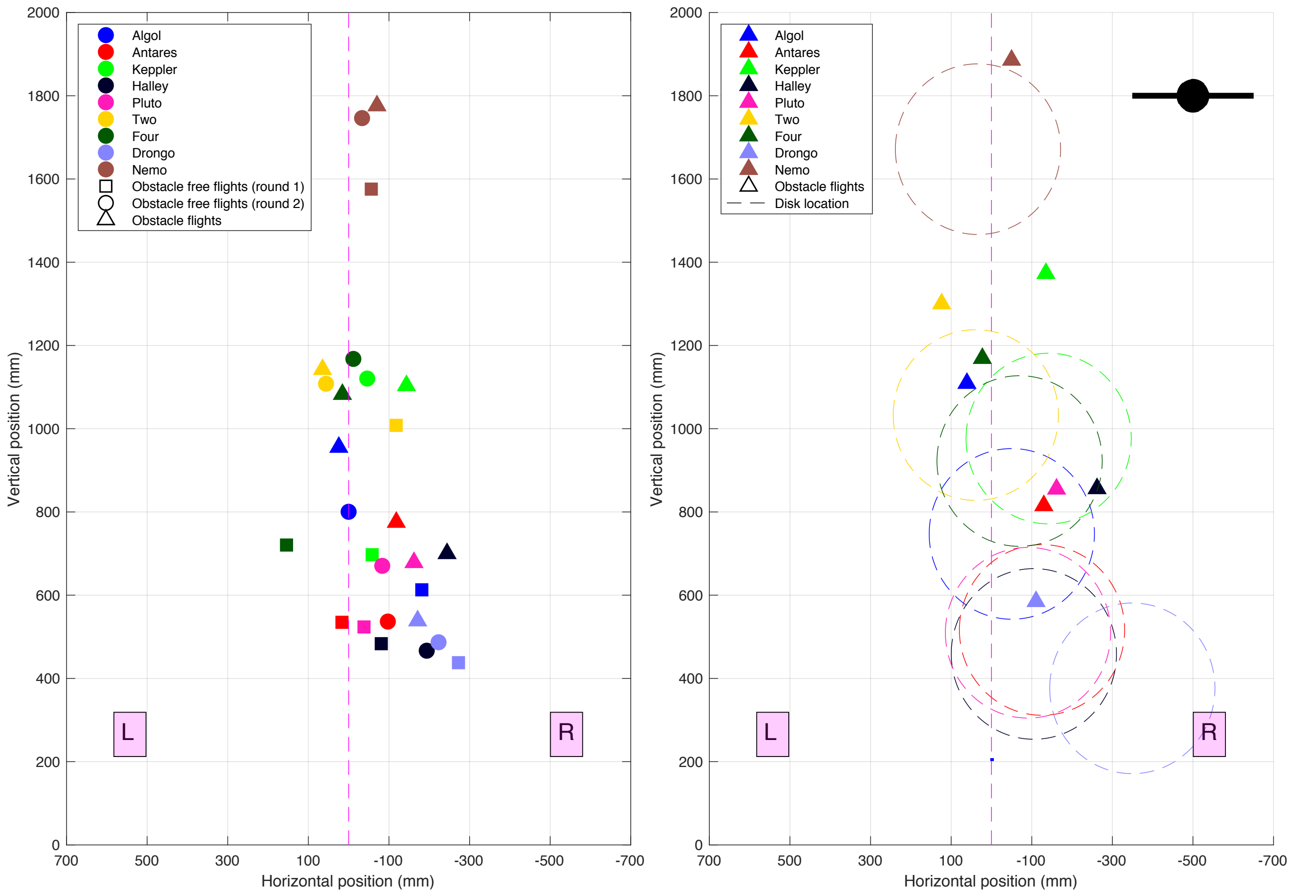

(a) (b)

Figure S4: (a) Mean position of each bird - averaged over the entire flight – for three experimental scenarios. Each colour represents an individual bird. The squares and circles denote the obstacle-free flights (Rounds 1 and 2, respectively), and the triangles denote the obstacle flights. (b) Comparison of the mean position of each bird in the cross section of the tunnel in the obstacle-free scenario (Round 2) and in the obstacle scenario. In each case the mean position represents an average over a 2 m flight segment spanning the obstacle (±1 m from the obstacle). The dashed circles represent the location of the obstacle for each bird. The centre of the circle represents the (local) mean position of each bird in Round 2 of the obstacle-free flights. The solid black circle represents the mean body size (3.96 cm) of the 9 birds and the thick black horizontal bar on either side of the circle depicts the mean wing length (13.09 cm). The dashed vertical line (pink) depicts the centre of the tunnel. R and L denote positions to the right and left of the tunnel, respectively, from the bird's point of view.

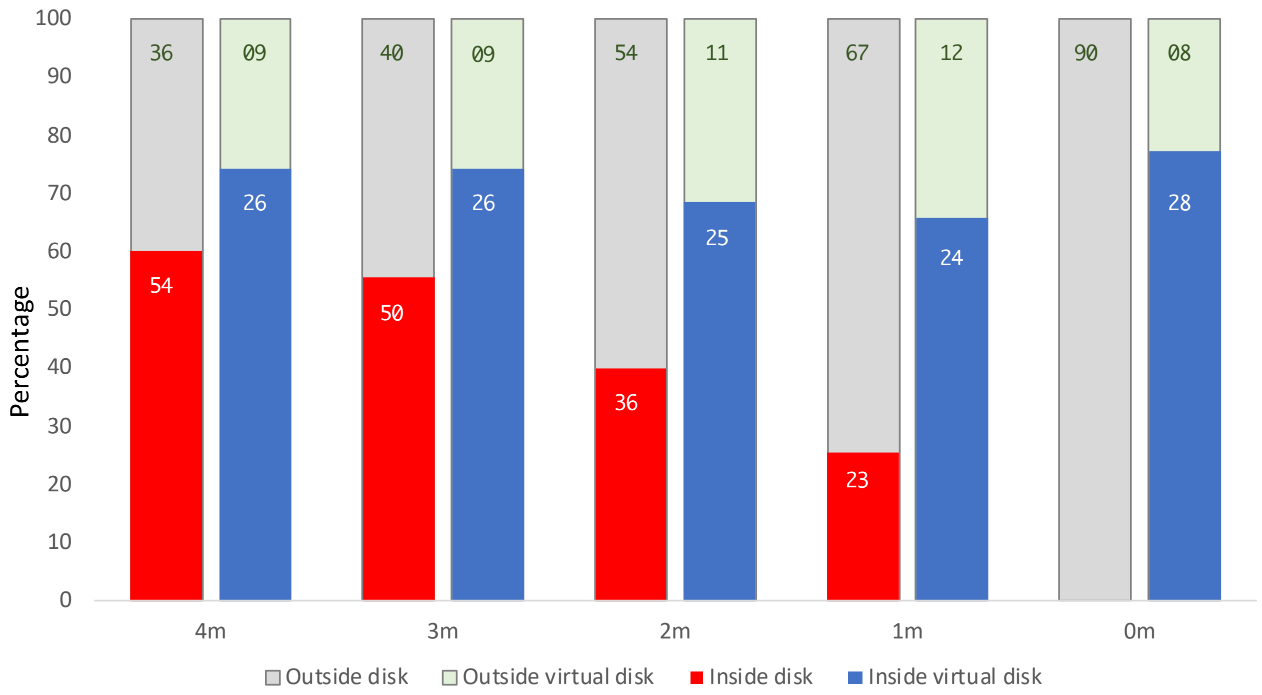

Figure S5: Percentage of flights that are on a collision course with the disk, at different axial distances from the disk. The red/grey columns show the numbers of flights that are within the projected area of the disk (i.e. on a collision course) or outside the projected area (i.e. not on a collision course). The blue/green columns show the corresponding figures for Round 2 of the obstacle-free scenario, for a 'virtual' disk placed at the position where it was located in the obstacle tests. Each bird performed 10 flights in the obstacle tests (totalling 90 flights), and 4 flights in Round 2 of the obstacle-free scenario (totalling 36 flights). Note that, in Round 2, the total number of flights is 35 at 4 m and 3 m, because only one flight was recorded for *Pluto* after the 3 m mark.

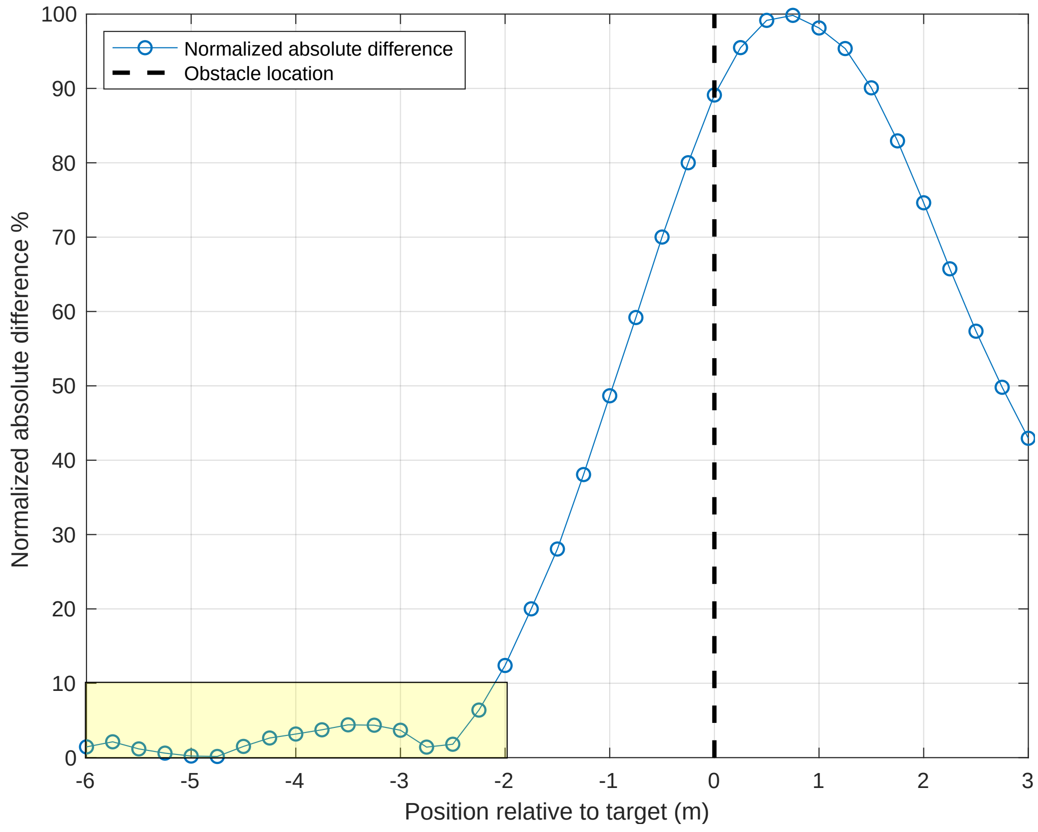

Figure S6: Normalised absolute difference between the mean radial distance profiles for the obstacle-free flights (Round 2) and the obstacle flights. The vertical dashed line depicts the position of the obstacle, and the yellow rectangle highlights the region in which the two profiles differ by less than 10%.

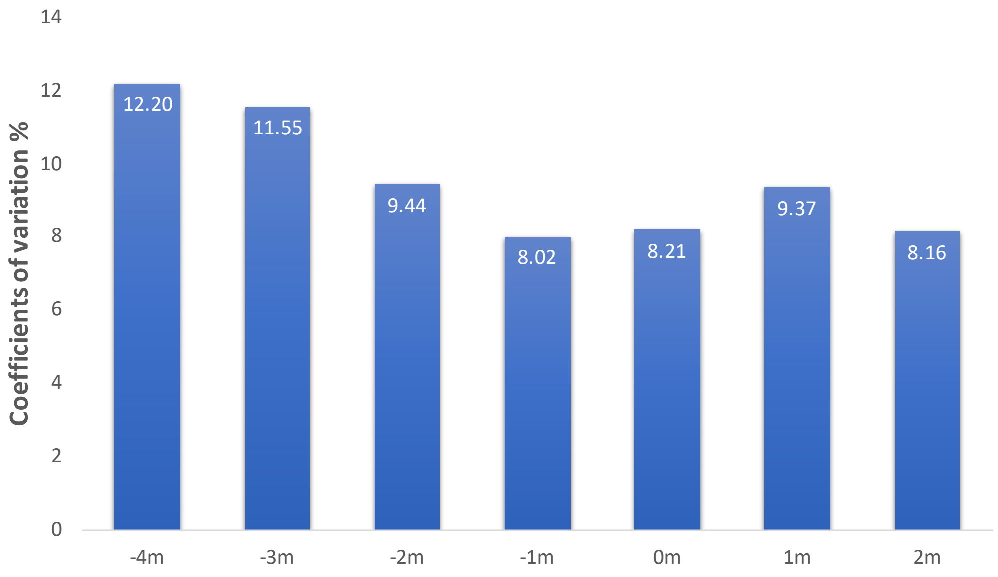

Figure S7: Mean coefficient of variation of flight speed (%), computed as in Table S2, at various axial distances from the obstacle.

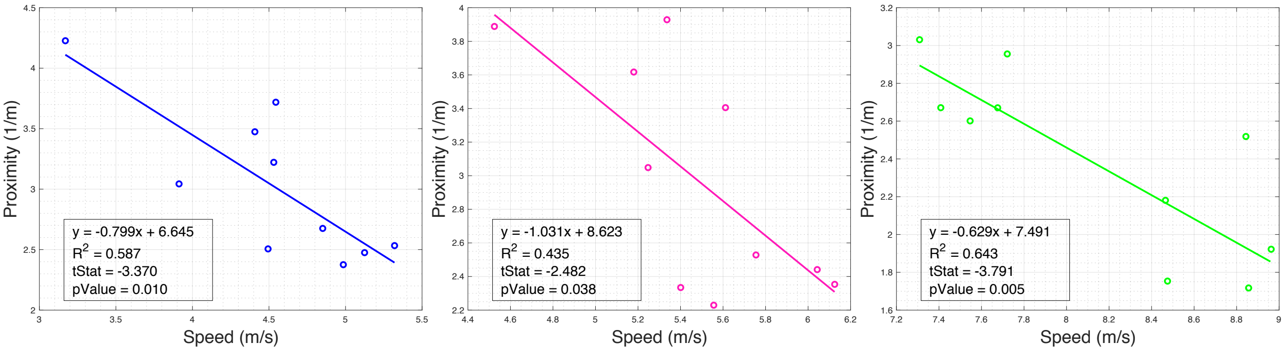

(a) Nemo (b) Drongo (c) Antares

Figure S8: Examples of correlation between flight speed and radial proximity to the obstacle at the point of crossing the obstacle, for those birds where the correlation is significant ($p < 0.05$).

2 Supplementary Tables

Table S1: Body measurements of each bird, including the left wing, right wing, body width, and weight.

	**Weight (g)**	**Left Wing (cm)**	**Right Wing (cm)**	**Body width (cm)**
**Algol**	46.00	12.90	12.70	4.10
**Anatres**	44.70	13.40	13.10	4.00
**Keppler**	38.90	13.00	13.50	4.10
**Halley**	47.40	11.90	13.10	3.40
**Pluto**	38.00	12.60	12.50	3.70
**Two**	50.90	13.20	12.60	3.80
**Four**	51.20	13.40	13.40	3.90
**Drongo**	37.10	13.40	13.30	4.00
**Nemo**	48.00	14.00	13.60	4.60
**MEAN**	**44.69**	**13.09**	**13.09**	**3.96**

Table S2: Mean Flight speed, Radial separation, Proximity, and Optic flow, and their coefficients of variation at the point of crossing the disk, for all birds.

	Flight speed (m/s)			Radial separation (m)			Proximity (1/m)			Optic flow (rad/s)		
	**MEAN**	**SD**	**CV**	**MEAN**	**SD**	**CV**	**MEAN**	**SD**	**CV**	**MEAN**	**SD**	**CV**
**Algol**	7.32	0.33	0.04	0.57	0.16	0.29	1.91	0.56	0.29	12.70	3.01	0.24
**Antares**	8.13	0.66	0.08	0.44	0.09	0.20	2.33	0.43	0.18	16.34	1.74	0.11
**Keppler**	6.68	0.62	0.09	0.83	0.15	0.18	1.24	0.24	0.19	8.06	1.74	0.22
**Halley**	6.54	0.48	0.07	0.45	0.14	0.31	2.53	1.09	0.43	14.12	4.47	0.32
**Pluto**	7.34	0.51	0.07	0.49	0.12	0.24	2.13	0.51	0.24	13.90	2.57	0.19
**Two**	4.38	0.25	0.06	0.52	0.05	0.10	1.95	0.22	0.11	7.87	0.68	0.087
**Four**	4.06	0.38	0.09	0.60	0.24	0.40	1.95	0.81	0.41	7.05	2.06	0.29
**Drongo**	5.48	0.46	0.08	0.37	0.08	0.21	2.83	0.61	0.21	13.74	2.13	0.16
**Nemo**	4.53	0.63	0.14	0.34	0.06	0.18	3.01	0.58	0.19	11.76	1.43	0.12
**MEAN**	**6.05**	**0.48**	**0.08**	**0.51**	**0.12**	**0.24**	**2.21**	**0.56**	**0.25**	**11.72**	**2.20**	**0.19**

Table S3: Covariance between the flight speed and the proximity, computed as the difference between the mean value of the optic flow and the product of the mean values of the flight speed and proximity, extracted from Table S2. In the formula for the optic flow, s_i and p_i refer to the speed and proximity for flight i ($i = 1, ..10$).

	**Mean flight speed (S)**	**Mean proximity (P)**	**Mean optic flow (O)** $= \sum_{i=1}^{10} \frac{s_i \times p_i}{10}$	**Product of mean flight speed and mean proximity** $(S \times P)$	**Covariance between S & P** $= O - (S \times P)$	**Covariance polarity**
Algol	7.32	1.91	12.7	13.95	-1.25	Negative
Anatres	8.13	2.33	16.34	18.9	-2.57	Negative
Keppler	6.68	1.24	8.06	8.26	-0.19	Negative
Halley	6.54	2.53	14.12	16.57	-2.44	Negative
Pluto	7.34	2.13	13.88	15.66	-1.78	Negative
Two	4.38	1.95	7.87	8.51	-0.65	Negative
Four	4.06	1.95	7.05	7.95	-0.89	Negative
Drongo	5.48	2.83	13.74	15.49	-1.75	Negative
Nemo	4.53	3.01	11.76	13.63	-1.87	Negative
**MEAN**	**6.05**	**2.21**	**11.72**	**13.36**	**-1.63**	**Negative**

Table S4: Mean and standard deviation of the angular position at which each bird passes the disk. 0 deg represents the vertically upward direction, and clockwise angles are positive.

	**Mean (deg)**	**SD (deg)**
**Algol**	-0.57	12.65
**Antares**	11.82	18.28
**keppler**	2.73	11.42
**Halley**	11.05	22.32
**Pluto**	14.39	17.09
**Two**	-5.039	12.67
**Four**	7.83	16.42
**Drongo**	-39.89	11.13
**Nemo**	-2.36	8.22

3 Captions for Supplementary Videos

Individual birds display distinct flight paths. We investigated the robustness of this behaviour by interposing a disk-shaped obstacle in the birds' paths. We found that each bird maintained its preferred flight path, veering away only minimally to avoid a collision.

Video S1

Comparison of a flight trajectory of bird *Drongo* while flying in an empty tunnel (pink), and while avoiding an obstacle, which was positioned in the bird's preferred flight path (white).

Video S2

Comparison of a flight trajectory of bird *Nemo* while flying in an empty tunnel (pink), and while avoiding an obstacle, which was positioned in the bird's preferred flight path (white).

Video S3

Four superimposed trajectories of bird *Drongo*, filmed from one of the front cameras while avoiding the obstacle. The bird shows a strong tendency to retain its preferred flight path over most of the trajectory, even after several trials with the intervening obstacle.

Video S4

Four superimposed trajectories of bird *Drongo*, filmed from one of the rear cameras while avoiding the obstacle. The bird shows a strong tendency to retain its preferred flight path over most of the trajectory, even after several trials with the intervening obstacle.

Video S5

Four superimposed trajectories of bird *Nemo*, filmed from one of the front cameras while avoiding the obstacle. The bird shows a strong tendency to retain its preferred flight path over most of the trajectory, even after several trials with the intervening obstacle.

Video S6

Four superimposed trajectories of bird *Nemo*, filmed from one of the rear cameras while avoiding the obstacle. The bird shows a strong tendency to retain its preferred flight path over most of the trajectory, even after several trials with the intervening obstacle.
